# Effects of *Physalis philadelphica* Lam. Husk Infusion on Obesity and Associated Metabolic Disturbances in an Experimental Model

**DOI:** 10.3390/ijms27146500

**Published:** 2026-07-22

**Authors:** Claudia I. Gamboa-Gómez, Jazel Barragán-Zúñiga, María Inés Guerra-Rosas, José Luis Gónzalez, Víctor Iván Sayago-Monreal, Cynthia P. Castillo-López, Karla F. Valles-Araiza, Adrián Alvarado-Aguilar, Juliana Morales-Castro, Martha Rodríguez-Morán, Fernando Guerrero-Romero

**Affiliations:** 1 Unidad de Investigación Biomédica del Instituto Mexicano del Seguro Social, Canoas 100, Durango 34067, Mexico; cpaolacl@gmail.com (C.P.C.-L.); alvaraguiadrian95@gmail.com (A.A.-A.); guerrero.romero@gmail.com (F.G.-R.); 2 Centro Estatal de Cancerología, Secretaria de Salud Durango, Av. 5 de Febrero esq, Antonio Norman Fuentes S/N, Zona Centro, Durango 34000, Mexico; ljbarraganz@gmail.com; 3 Laboratorio Nacional Conahcyt de Apoyo a la Evaluación de Productos Bioticos (LaNAEPBi), Instituto Tecnológico de Durango, Tecnológico Nacional de México (TecNM), Blvd. Felipe Pescador 1830 Ote, Colonia Nueva Vizcaya, Durango 34080, Mexico; m.guerra@itdurango.edu.mx (M.I.G.-R.); victor_sayago95@hotmail.com (V.I.S.-M.); 24041584@itdurango.edu.mx (K.F.V.-A.); morales@itdurango.edu.mx (J.M.-C.); 4 Departamento de Patología, Hospital General de Zona No. 1, Instituto Mexicano del Seguro Social, Canoas S/N, Durango 34067, Mexico; jlgpatologia@yahoo.com.mx

**Keywords:** herbal infusions, obesity, oxidative stress, impaired glucose

## Abstract

Phytotherapy has emerged as a promising strategy for the management of obesity and related metabolic disorders. In this context, species of the *Physalis* genus have been widely recognized in traditional medicine for their potential therapeutic effects against these conditions. This study aimed to evaluate the effects of *Physalis philadelphica* husk infusion on obesity and associated metabolic disturbances such as hyperglycemia, insulin resistance, hepatic steatosis, and oxidative stress markers in diet-induced obese rats. Male Wistar rats were allocated into three groups (n = 8): (1) healthy control, (2) obese control group, and (3) intervention group (husk infusion). Obesity was induced using a high-fructose and high-saturated fat diet (~511.1 kcal/100 g), followed by 16 weeks of husk infusion administration. After euthanasia, hyperglycemia, insulin resistance, hepatic steatosis, and oxidative stress markers were analyzed. Results showed that rats receiving the husk infusion exhibited a significant reduction in weight gain (~7.2%), serum glucose levels (22.8%), and free fatty acids (26%) compared to the obese control group, while no significant effects on insulin sensitivity, hepatic steatosis, or several lipid-related parameters were observed. Regarding oxidative stress markers, serum nitrite levels decreased by 33%, and malondialdehyde (MDA) levels in the liver (30%) and adipose tissue (38%) were significantly reduced compared to the obese control group. Moreover, treated rats showed a significant increase in catalase (~1.2-fold) and superoxide dismutase (~2.8-fold) activity in the liver. These findings suggest that *Physalis philadelphica* husk infusion reduces hyperglycemia and improves oxidative stress in obese rats.

## 1. Introduction

Obesity is a growing global health problem that currently affects more than one billion people worldwide. Its increasing prevalence is associated with a higher risk of noncommunicable diseases and millions of obesity-related deaths each year [1]. Obesity results from an imbalance between energy intake and expenditure, leading to excessive adipose tissue (AT) expansion. This pathological expansion is associated with dysregulated lipolysis and an increased release of free fatty acids (FFAs) into the circulation [2], disrupting lipid homeostasis and promoting lipotoxicity [3]. Elevated circulating FFAs contribute to insulin resistance and pancreatic β-cell dysfunction by altering fatty acid metabolism and intracellular signaling pathways. These metabolic disturbances impair insulin action in key target tissues, including skeletal muscle and the liver, thereby exacerbating systemic metabolic dysfunction [4].

Furthermore, chronic lipid overload promotes the accumulation of ectopic fat in non-adipose tissues such as skeletal muscle, the pancreas, and the liver. Excess lipid deposition in these organs can impair the function of cellular organelles, including mitochondria, the endoplasmic reticulum, and lysosomes, leading to oxidative stress and activation of inflammatory pathways through increased production of reactive oxygen species (ROS) and pro-inflammatory mediators [5]. Although cells possess endogenous antioxidant defense mechanisms, including enzymes such as superoxide dismutase (SOD) and catalase (CAT), excessive ROS production can overwhelm these protective systems, resulting in oxidative stress [6].

Oxidative stress is considered a key mechanistic link between obesity and its associated metabolic complications. Excess adiposity promotes ROS generation through multiple biochemical pathways, including NADPH oxidase activation, glyceraldehyde auto-oxidation, mitochondrial oxidative phosphorylation, protein kinase C (PKC) activation, and the polyol and hexosamine pathways. In turn, oxidative stress exacerbates metabolic dysfunction by promoting adipocyte hypertrophy, adipogenesis, chronic low-grade inflammation, and insulin resistance, thereby contributing to the progression of obesity and its related comorbidities [7]. The combined effects of lipotoxicity, insulin resistance, chronic inflammation, and oxidative stress make the liver particularly susceptible to metabolic injury. Consequently, persistent disruption of metabolic homeostasis in obesity may ultimately result in liver damage, including hepatic steatosis. Hepatic steatosis, considered the earliest stage of metabolic dysfunction-associated steatotic liver disease, is characterized by excessive triglyceride (TG) accumulation within hepatocytes, typically exceeding 5% of liver tissue, and is strongly associated with obesity, insulin resistance, and dysregulated lipid metabolism [8].

Phytotherapy offers a promising approach to address obesity and related metabolic disorders, and species of the *Physalis* genus have attracted attention for their well-documented antioxidant properties. For example, Pino-de la Fuente et al. (2020) [9] demonstrated that daily consumption of 300 mg/kg of *Physalis peruviana* L. fresh pulp improved hepatic lipoperoxidation and insulin resistance in obese mice. The authors attributed these beneficial effects to the antioxidant activity of *P*. *peruviana*, which protected skeletal muscle from oxidative damage and reduced lipid peroxidation. By limiting excessive ROS production, this antioxidant effect may preserve insulin signaling pathways and mitigate the impairment of insulin sensitivity associated with obesity.

More recently, Moussa et al. (2022) [10] reported that *Physalis peruviana* attenuated AT hypertrophy and oxidative stress in obese rats. The authors suggested that these beneficial effects were mediated through the modulation of adipocyte-derived hormones (adipokines) and the free radical-scavenging activity of the fruit, thereby contributing to improved adipose tissue function and redox homeostasis.

Mexico is recognized as a center of diversity for the *Physalis* genus, harboring more than 70 species, many of which are endemic to the country [11]. Among these, *Physalis ixocarpa* and *Physalis philadelphica*, commonly known as husk tomato, green tomato, or tomatillo, are the most widely cultivated and consumed species in Mexico, the United States, and Central America [12]. Both species are diploid and share highly similar morphological characteristics, which have led to frequent taxonomic confusion, with some authors considering them synonymous species [13]. A distinctive feature of these fruits is the enlarged papery husk (calyx), which completely encloses the developing fruit and protects it from adverse environmental conditions, pests, and pathogens [14]. This protective structure is also associated with the accumulation of bioactive compounds, including polyphenols and other metabolites with antioxidant and repellent properties [15]. Despite being a rich source of phytochemicals with potential health-promoting effects, the husk is commonly discarded during processing and is, therefore, considered an agri-food by-product [16].

In traditional medicine, both species (*ixocarpa* and *philadelphica*) have been used as tonics, eyewashes, laxatives, diuretics, and remedies for respiratory and gastrointestinal disorders, bladder ulceration, inflammatory conditions, splenic inflammation, ascites, snake bites, and diabetes [17,18,19]. Previous in vitro studies have demonstrated that Mexican *Physalis* varieties possess significant antioxidant activity [20]. In addition, our research group has shown that husk infusions and extracts from *Physalis ixocarpa* exert acute hypoglycemic and antioxidant effects in both in vitro and in vivo models [21]. Furthermore, chronic administration of *Physalis philadelphica* husk extracts obtained by aqueous maceration and ultrasound-assisted extraction has been shown to improve hyperglycemia, insulin resistance, hepatic steatosis, and oxidative stress in diet-induced obese rats [22].

Despite these promising findings, an important knowledge gap remains. Although *P*. *philadelphica* husk infusion is the preparation traditionally used in Mexican folk medicine and represents the most common form of human consumption, its long-term effects on obesity-associated metabolic disturbances have not yet been evaluated in an experimental model. Moreover, the biological effects of crude infusions cannot be directly extrapolated from those of concentrated extracts because their phytochemical composition and the bioavailability of their bioactive compounds differ considerably.

Therefore, the novelty of the present study lies in evaluating, for the first time, the chronic effects of *P*. *philadelphica* husk infusion, a preparation that closely reflects its traditional medicinal use, on obesity and its associated metabolic alterations in an experimental model. By investigating the form in which this plant is traditionally consumed, rather than concentrated extracts, this study provides evidence of greater ethnopharmacological and translational relevance for the potential use of *P*. *philadelphica* in the management of obesity-associated metabolic disorders.

## 2. Results

Husk infusion pH was 6.0, while the yield was approximately 17.86%, equivalent to 1.9 mg of lyophilized material per mL of aqueous infusion.

Regarding the chemical characterization, the identification and quantification of bioactive compounds are presented in Table 1. The bioactive compounds with the highest concentrations were gallic acid, protocatechuic acid, vanillic acid, caffeic acid, and quercetin.

**Figure 1:**
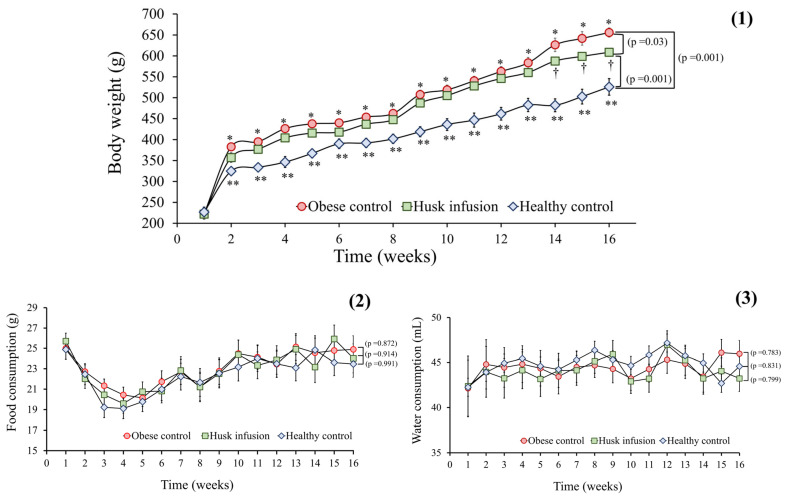
Body weight (**1**), food consumption (**2**), and water consumption (**3**) of obese rats that received a husk infusion of *Physalis philadelphica*. Values are presented as mean ± standard error. * *p* < 0.05 relates to the comparison between the Healthy control and Obese control, ** *p* < 0.05 between the Healthy control and Husk Infusion, and † *p* < 0.05 between the Obese control and Husk Infusion. Significance was determined using one-way ANOVA followed by Tukey’s post hoc test.

At baseline, all groups had comparable body weights (~222 g). After 16 weeks, the healthy group reached 525.87 ± 19.9 g, representing a 2.37-fold increase relative to baseline. In contrast, obese control rats reached 655.88 ± 10.9 g, corresponding to a 2.95-fold increase and a final body weight approximately 25% higher than that of the healthy group. Treatment with the husk infusion attenuated weight gain, resulting in a final body weight of 608.55 ± 5.5 g (2.74-fold increase), which was approximately 7.2% lower than that of the obese control group (Figure 1).

Food and water intake did not differ significantly among groups (Figure 1). Food consumption averaged ~25 g/day throughout the experimental period. However, because the diets differed in caloric density, estimated energy intake was higher in the obese control and husk infusion-treated groups than in the healthy control group. Based on average daily food consumption, healthy rats consumed approximately 93.1 kcal/day, whereas rats fed the high-fat/high-fructose diet consumed approximately 127.8 kcal/day. Thus, despite similar food intake by weight, the obese control and treated groups exhibited a higher daily energy intake than the healthy control group.

**Figure 2:**
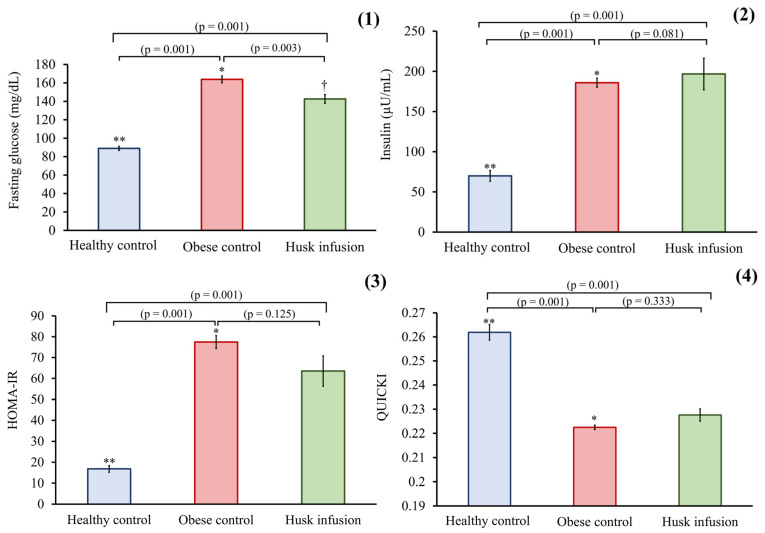
Fasting glucose (**1**), insulin (**2**), HOMA-IR (**3**), and QUICKI values (**4**) of obese rats that received a husk infusion of *Physalis philadelphica*. Values are presented as mean ± standard error. * *p* < 0.05 relates to comparison between the Healthy control and the Obese control, ** *p* < 0.05 between the Healthy control and Husk Infusion, and † *p* < 0.05 between the Obese control and Husk Infusion. Significance was determined using one-way ANOVA followed by Tukey’s post hoc test.

Regarding the assessment of obesity-associated metabolic disturbances, obese rats exhibited serum fasting glucose levels of 168.5 ± 2.0 mg/dL, insulin concentrations of 185.87 ± 5.7 µU/mL, Homeostatic Model Assessment (HOMA-IR) index values of 77.46 ± 3.1, and Quantitative Insulin Sensitivity Check Index (QUICKI) values of 0.2225 ± 0.0009 (Figure 2). Compared with healthy rats, obesity was associated with a 1.73-fold increase in fasting glucose, a 2.66-fold increase in insulin concentrations, and a 4.61-fold increase in HOMA-IR values, whereas QUICKI values were reduced by approximately 15%. In contrast, rats treated with the husk infusion showed a significant reduction in fasting glucose levels (22.8%), whereas insulin concentrations, HOMA-IR values, and QUICKI values did not differ significantly from those of the obese control group (Figure 2).

**Figure 3:**
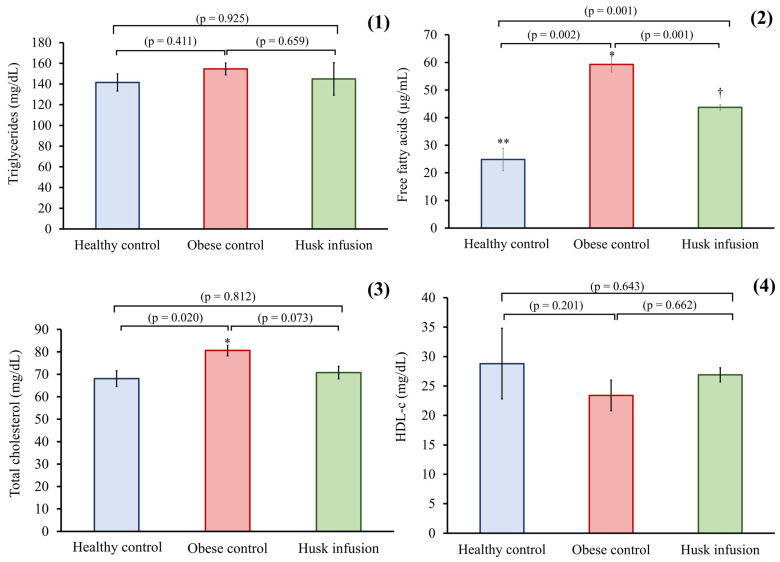
Serum triglycerides (**1**), free fatty acids (**2**), total cholesterol (**3**), and HDL-c (**4**) serum concentration of obese rats that received a husk infusion of *Physalis philadelphica*. Values are presented as mean ± standard error. * *p* < 0.05 relates to the comparison between the Healthy control and the Obese control, ** *p* < 0.05 between the Healthy control and Husk Infusion, and † *p* < 0.05 between the Obese control and Husk Infusion. Significance was determined using one-way ANOVA followed by Tukey’s post hoc test.

On one hand, no significant differences in serum TG or high-density lipoprotein cholesterol (HDL-c) levels were observed between groups (Figure 3). On the other hand, the obese control group showed serum FFAs levels of ~59.3 ± 2.8 µg/mL, which represents an increase of approximately 58% compared with the healthy control group. In contrast, obese rats treated with the husk infusion exhibited FFA levels of ~43.7 ± 2.8 µg/mL, corresponding to a significant reduction of about 26% relative to the obese control group (Figure 3). Regarding total cholesterol, levels remained within the normal range across all groups (Figure 3), with no significant alterations.

**Figure 4:**
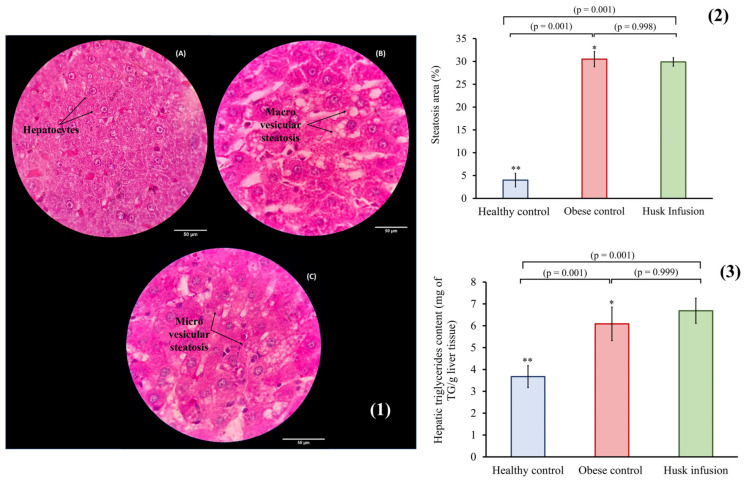
Representative histological sections of liver tissue (hematoxylin and eosin (H&E) staining, 100×) (**1**): healthy rats (**A**), obese control rats (**B**), and rats administered husk infusion (**C**). The figure also shows the percentage of steatosis area for all groups (**2**) and liver triglyceride content (**3**). Five images per section and ten fields per image were analyzed for each rat. Measurements were averaged to obtain a single value for each animal, and statistical analyses were performed using the individual animal as the experimental unit (n = 8 per group). Values are presented as mean ± standard error. * *p* < 0.05 relates to the comparison between the Healthy control and the Obese control, ** *p* < 0.05 between the Healthy control and Husk Infusion. Significance was determined using one-way ANOVA followed by Tukey’s post hoc test.

The results for hepatic TG content and steatosis are presented in Figure 4. Compared with the healthy control group (Figure 4(1A)), the obese control group exhibited marked lipid droplet accumulation in hepatic tissue (Figure 4(1B)). Likewise, the treated group showed a steatotic pattern comparable to that observed in the obese control group, with no significant reduction in the steatosis area (Figure 4(1C)). Histological evaluation revealed grade 1 steatosis, characterized by macrovesicular and microvesicular lipid accumulation occupying approximately 30% of the hepatic parenchyma, excluding unstained regions. These histological findings were consistent with hepatic TG quantification, which revealed no significant differences between the obese control and the treated groups (Figure 4).

The results for oxidative stress markers, specifically for end products of nitric oxide or nitrites (NO_end-PD_) and malondialdehyde (MDA), are presented in Table 2. Compared with healthy rats, obese control rats exhibited a 2.48-fold increase in serum NO_end-PD_, a 1.96-fold increase in liver NO_end-PD_, and a 1.85-fold increase in adipose tissue NO_end-PD_. Likewise, MDA levels were markedly elevated, showing a 5.71-fold increase in serum, a 2.67-fold increase in the liver, and a 5.05-fold increase in adipose tissue. In contrast, treatment with the husk infusion reduced NO_end-PD_ levels by approximately 33% in serum and 29% in the liver compared with the obese control group. Additionally, husk infusion decreased MDA levels by 19% in serum, 30% in the liver, and 38% in adipose tissue.

**Figure 5:**
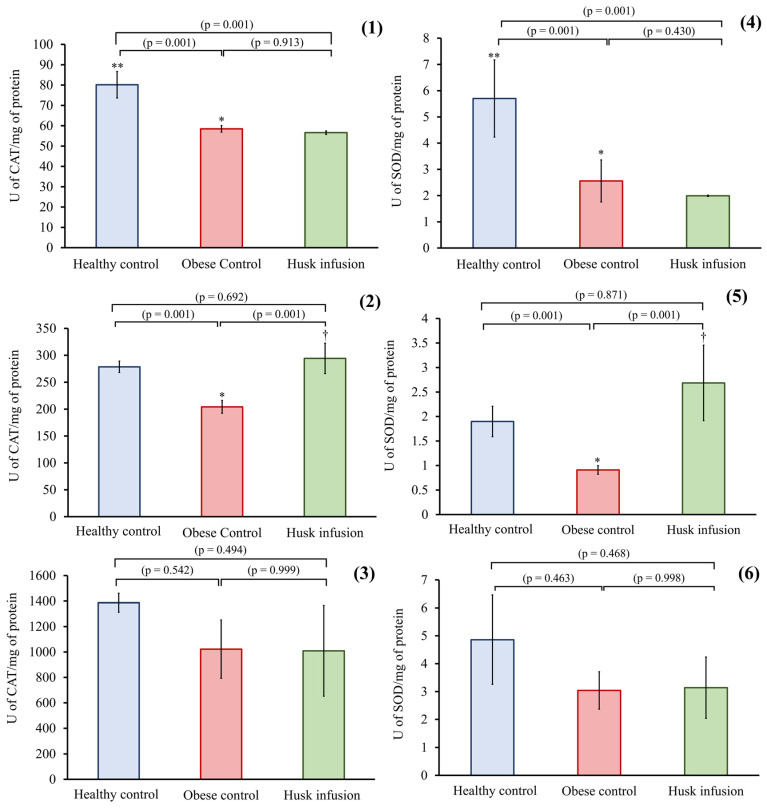
Catalase (CAT) in serum (**1**), liver (**2**) and adipose tissue (**3**). Superoxide dismutase (SOD) in serum (**4**), liver (**5**) and adipose tissue (**6**) of obese rats that received a husk infusion of *Physalis philadelphica*. Values are presented as mean ± standard error. * *p* < 0.05 relates to the comparison between the Healthy control and the Obese control, ** *p* < 0.05 between the Healthy control and Husk Infusion, and † *p* < 0.05 between the Obese control and Husk Infusion. Significance was determined using one-way ANOVA followed by Tukey’s post hoc test.

Regarding antioxidant enzyme activity, the results are shown in Figure 5. In serum, CAT activity was significantly lower in obese control rats (58.4 ± 0.6 U/mg protein) than in healthy controls (80.2 ± 1.6 U/mg protein). A similar pattern was observed in the liver, where CAT activity decreased from 289.3 ± 1.6 U/mg protein in healthy rats to 207.3 ± 0.6 U/mg protein in obese controls.

A similar trend was observed for SOD activity. In serum, healthy rats exhibited 5.7 ± 1.6 U/mg protein, whereas obese control rats showed significantly lower activity (2.5 ± 0.6 U/mg protein). Likewise, hepatic SOD activity decreased from 1.8 ± 1.6 U/mg protein in healthy controls to 0.9 ± 0.6 U/mg protein in obese control rats. In contrast, no significant differences in CAT or SOD activity were observed in adipose tissue among the experimental groups.

In rats receiving husk infusion, hepatic CAT activity increased significantly compared with the obese control group, reaching 252.5 ± 1.1 U/mg protein (approximately 1.2-fold higher). Likewise, hepatic SOD activity increased to 2.7 ± 1.1 U/mg protein (approximately 2.8-fold higher than the obese control group). No significant changes were observed in serum or adipose tissue antioxidant enzyme activities following husk infusion administration (Figure 5).

## 3. Discussion

Our results demonstrate that the administration of husk infusion exerts a positive effect on obesity through weight gain reduction, and by reducing hyperglycemia and oxidative stress in diet-induced obese rats.

Administration of the husk infusion resulted in approximately a 7.2% reduction in body weight compared with the obese control group. This finding is biologically relevant, as a sustained body weight reduction of 5–10% has been associated with clinically meaningful improvements in obesity-related metabolic alterations [23]. Notably, the anti-obesity effect of the husk infusion occurred independently of food and water intake, as neither parameter differed significantly among the experimental groups throughout the intervention. Therefore, the reduction in body weight gain cannot be attributed to decreased food or fluid consumption.

Although we do not investigate the underlying molecular mechanisms involved in this effect, we hypothesize that the reduction in body weight may be partially explained by the decrease in intestinal absorption of carbohydrates. This hypothesis is supported by our previous studies, in which *Physalis* husk infusion inhibited, in vitro, the activity of the digestive enzymes α-amylase and α-glucosidase by approximately 90% and, in vivo, reduced carbohydrate absorption by up to 10% compared with the negative control [19]. These findings suggest that inhibition of carbohydrate digestion and absorption may contribute to the lower body weight gain observed in the present study. Furthermore, the phytochemical profile of the husk infusion may also have contributed to its anti-obesity effects. Among the identified compounds, protocatechuic acid and caffeic acid have been reported to attenuate body weight gain and adiposity through multiple mechanisms, including the inhibition of carbohydrate and lipid absorption, an increase in fatty acid oxidation [22], promotion of cellular thermogenesis [23], and attenuation of obesity-associated oxidative stress [24]. Therefore, the anti-obesity effect observed in the present study is likely the result of the combined action of these bioactive compounds rather than a single mechanism. Further studies are required to identify the specific molecular pathways responsible for the anti-obesity effects of *P*. *philadelphica* husk infusion.

Our findings indicate that husk infusion administration significantly reduced fasting glucose levels in obese rats. These results are consistent with previous studies reporting the hypoglycemic effects of *Physalis* husk preparations [19,25]. The observed reduction in glucose levels may be associated, at least in part, with the presence of bioactive compounds identified in the infusion, particularly phenolic acids such as gallic, protocatechuic, vanillic, and caffeic acids, as well as the flavonoid quercetin. These compounds have previously been reported to exert glucose-lowering effects in experimental models [26,27,28]. Additionally, although present at lower concentrations, chlorogenic acid, rutin, and luteolin may also have contributed to the observed response, as these phytochemicals have been associated with hypoglycemic, antioxidant, and anti-inflammatory activities [29,30,31].

Interestingly, the reduction in fasting glucose occurred without significant changes in fasting insulin concentrations or indices of insulin resistance. This finding suggests that the hypoglycemic effect of the husk infusion may involve mechanisms independent of improvements in systemic insulin sensitivity. Previous studies have shown that several polyphenolic compounds can activate AMP-activated protein kinase (AMPK), thereby enhancing glucose uptake in peripheral tissues and suppressing hepatic gluconeogenesis. Furthermore, interactions between dietary polyphenols and the gut microbiota have been associated with increased secretion of glucagon-like peptide-1 (GLP-1), which contributes to glucose homeostasis [32,33]. Although these mechanisms were not evaluated in the present study, they represent plausible pathways through which husk infusion may exert its glucose-lowering effects. Further studies are required to confirm these hypotheses and elucidate the molecular mechanisms involved.

Our results also showed that the husk infusion significantly reduced serum FFA concentrations. Elevated circulating FFAs are closely associated with obesity, insulin resistance, and ectopic lipid accumulation; therefore, their reduction suggests an improvement in lipid metabolism. Although the molecular mechanisms underlying this effect were not investigated in the present study, the phytochemical composition of the husk infusion may partially explain these findings. For instance, quercetin, one of the bioactive compounds identified in the infusion, has been reported to promote lipophagy and enhance mitochondrial β-oxidation of fatty acids through activation of the AMP-activated protein kinase (AMPK) signaling pathway [34]. Likewise, ferulic acid has been shown to regulate hepatic lipid metabolism by downregulating the expression of key lipogenic genes, including sterol regulatory element-binding protein-1c (SREBP-1c), fatty acid synthase (FAS), and acetyl-CoA carboxylase (ACC), while upregulating carnitine palmitoyltransferase-1A (CPT1A), a rate-limiting enzyme involved in mitochondrial fatty acid β-oxidation [35]. Collectively, these mechanisms may have contributed to the reduction in circulating FFAs concentrations observed in the present study. Nevertheless, further studies are required to elucidate the molecular pathways involved and to confirm whether these mechanisms are responsible for the metabolic effects of *P*. *philadelphica* husk infusion.

Despite the significantly reduced serum FFA concentrations, no significant improvements in hepatic TG content or hepatic steatosis were observed following husk infusion administration. This discrepancy may be explained by the fact that hepatic lipid accumulation is a multifactorial process involving not only circulating FFAs but also de novo lipogenesis, fatty acid uptake, lipid export, and fatty acid oxidation. Therefore, although the husk infusion reduced circulating FFAs, this effect may not have been sufficient to reverse the hepatic lipid accumulation established after prolonged exposure to the obesogenic diet. Additionally, the degree of steatosis observed in the present study was relatively mild (grade 1), which may have limited the detection of further histological improvements. Further studies are needed to determine whether longer intervention periods or higher doses of the infusion could exert beneficial effects on hepatic lipid accumulation.

Oxidative stress is recognized as a key mechanism underlying the development of obesity-associated metabolic disorders, as excessive production of ROS promotes lipid peroxidation, disrupts insulin signaling, and contributes to insulin resistance. Previous studies on *Physalis* species have demonstrated that their nutraceutical potential is largely attributed to their antioxidant properties. Interventions with *Physalis* have been reported to protect skeletal muscle from oxidative damage and reduce lipid peroxidation, thereby limiting excessive ROS production [9,10]. Consistent with these findings, the present study demonstrated that *P*. *philadelphica* husk infusion significantly reduced nitrite concentrations in both the serum and liver, as well as MDA levels in the liver and adipose tissue, indicating attenuation of systemic and tissue oxidative stress. Furthermore, the infusion significantly increased the activity of the antioxidant enzymes CAT and SOD in the liver, whereas no significant changes were observed in adipose tissue. The tissue-specific antioxidant responses observed in this study may be explained by differences in the bioavailability of bioactive compounds, tissue metabolism, or the intrinsic antioxidant capacity of each organ. Moreover, the antioxidant effects of the husk infusion are likely associated with its polyphenol content. Polyphenols such protocatechuic acid, ferulic acid and quercetin exert antioxidant activity through multiple complementary mechanisms, including direct scavenging of reactive oxygen species, inhibition of lipid peroxidation, chelation of transition metal ions, and modulation of endogenous antioxidant defense systems [36,37,38]. Collectively, these mechanisms may contribute to the reduction in oxidative stress observed in the present study and, consequently, to the improvement of obesity-associated metabolic alterations.

Moreover, the differences in antioxidant efficacy among the serum, liver, and AT could be explained by tissue-specific variations in the bioavailability of these compounds. Notably, many of the bioactive compounds identified in the husk infusion are esters, glycosides, or polymers [18], which require hydrolysis by intestinal enzymes and/or the gut microbiota before they can be absorbed and become bioavailable [38]. Therefore, the great antioxidant effect observed in the liver could be explained by the fact that, once absorbed and conjugated in the small intestine, these compounds undergo hepatic glucuronidation, sulfation, or methylation, whereas the antioxidant activity observed in other tissues, such as AT, could be partly related to the reabsorption of the above-mentioned bioactive compounds [38]. Undoubtedly, further studies in the field are required.

### Limitations of the Study

Several limitations should be acknowledged. First, only a single dose of the infusion was evaluated. This dose was selected to mimic the consumption of one cup (240 mL), which represents a common serving size in traditional use [39]. Therefore, dose–response relationships could not be established. Second, only male animals were included in the study. Although this approach is commonly used in preclinical research to reduce biological variability and facilitate the characterization of treatment effects, potential sex-specific responses cannot be excluded. Third, inflammation-related outcomes were not assessed; thus, the contribution of inflammatory pathways to the observed metabolic effects remains unclear. Fourth, although body weight was monitored weekly throughout the experimental period, the data were not analyzed using longitudinal repeated-measures statistical models, which may provide a more comprehensive assessment of body weight trajectories over time. Finally, the molecular mechanisms underlying the hypoglycemic, antioxidant, and anti-obesity effects of *P*. *philadelphica* husk infusion were not directly investigated. Further studies are needed to identify the signaling pathways and molecular targets involved in these biological responses.

## 4. Materials and Methods

### 4.1. Plant Material and Infusion Preparation

The husk of *P*. *philadelphica* was obtained from the Francisco Villa supply market in Durango, Mexico, during the spring–summer season of 2022. The husks were stored in a dark room for 3 days at room temperature (25 °C). The taxonomic identification was performed by the Interdisciplinary Research Center for Regional Integral Development of the National Polytechnic Institute, Durango Unit, Mexico (CIIDIR) herbarium, and the reference specimen was No. 61091. Additionally, the plant name was verified in the World Flora Online database (http://www.worldfloraonline.org) on 22 February 2022.

The husk infusion was prepared using a 1:100 (*w*/*v*) ratio of ground, dried plant material to solvent. This concentration was selected to reflect the typical intake of individuals who regularly consume herbal teas [40], thereby ensuring the relevance and applicability of the findings to common dietary habits.

Specifically, 1 g of the sample was mixed with 100 mL of distilled water heated to 96 °C and allowed to steep for 10 min. The resulting infusion was filtered twice through a 0.5 mm pore size filter, and its pH was measured using a pH meter (Beckman Coulter Inc., Fullerton, CA, USA). Subsequently, samples were lyophilized using a FreeZone Console Freeze Dry System (Ulysses, KS, USA).

### 4.2. Solid Yields and Chemical Characterization

The infusion yield percentage (Y%) was calculated using the following equation:

Y% = [W_1_ (g)/W_2_ (g)] × 100% (1)

W_1_ is the weight of the corresponding lyophilized infusion, and W_2_ is the weight of dried husk.

Results are reported as the means of two independent preparations.

The method described by Guerrero-Romero et al. [19] was used to perform chemical characterization. A Waters Corporation Acquity UPLC system (Milford, MA, USA) coupled to a tandem Xevo TQ-S triple quadruple mass spectrometer was utilized. The UPLC system included a sample manager operating at a temperature of 5 °C and a quaternary solvent manager. A column of Acquity UPLC BEH C_8_ with a particle size of 1.7 μm and dimensions of 100 mm *×* 2.1 mm, maintained at a temperature of 30 °C, was used to identify the bioactive compounds. The analytical software MassLynx v. 4.1 (Waters Corp., Milford, MA, USA) was employed for system management and data analysis.

The method was validated following the procedure described in the International Conference on Harmonization guidelines [41]. The specificity of the method was ascertained by analyzing standard compounds and samples.

### 4.3. Experimental Animals

The animal study was carried out in accordance with the regulations established by the National Institutes of Health [42] and with the Norma Oficial Mexicana [43]. Moreover, the experimental procedure was registered and authorized by the Research Committee of the Mexican Social Security Institute (F-CNIC-2023-048).

Male Wistar rats (N = 24), aged twelve weeks and weighing 220 ± 20 g (Universidad Autónoma de México, Campus Juriquilla, Querétaro, México) were maintained in an animal laboratory with a 12–12 h light–dark cycle and a temperature of 26 ± 1 °C. Rodents were acclimatized for a week before the start of the experiments with a rodent diet (Rodent Lab Chow 5001, Purina^®^, Québec, QC, Canada) (~372.5 Kcal/100 g, consisting of 4.5% lipids, 60% carbohydrates, and 23% proteins).

For obesity induction, a high fructose and saturated fat diet was used: 15.1% of lipids (wherein 27% of them were saturated fat from lard), 70.9% of carbohydrates (wherein 45.6% of them were corn crystalline fructose), and 13.9% of proteins. The caloric content of the diet was approximately 511.1 Kcal/100 g.

The experimental groups (n = 8) were as follows: (1) a healthy control group receiving a standard rodent diet, (2) an obese control group receiving an obesogenic diet, and (3) a group receiving an obesogenic diet plus husk infusion.

The treated group received the husk infusion by oral *gavage* once daily in the morning. The infusion was freshly prepared each day prior to administration. The selected dose was based on the consumption of one cup of infusion (240 mL), which is a commonly consumed serving size [39]. Additionally, we previously reported that this dose of husk infusion exerts acute hypoglycemic and antioxidant effects in both in vitro and in vivo models [19]. For dose extrapolation, a daily intake of one cup of infusion (240 mL) by a 60 kg adult was considered, corresponding to a human equivalent dose (HED) of 4 mL/kg body weight (240 mL/60 kg = 4 mL/kg). The animal dose (AD) was estimated according to the dose conversion approach proposed by Reagan-Shaw et al. [44]:

HED = AD × (animal Km/human Km)(2)

where the Km values for rats and adult humans are 6 and 37, respectively. Therefore,

AD = HED/(animal Km/human Km)

AD = 4/(6/37)

AD = 24.69 mL/kg body weight

Considering that the lyophilized infusion contained approximately 1.95 mg of solids per mL of infusion, the administered dose corresponded to 48.15 mg of lyophilized solids per kg body weight (24.69 mL/kg × 1.95 mg/mL = 48.15 mg/kg).

Since the animals’ weights increase throughout the experiment, the dose was adjusted weekly.

The healthy rats and obese control groups received an inert vehicle (drinking water) via *gavage* once daily in the morning, at the same volume as the infusion administered to the intervention group.

Throughout the 16-week trial period, the rats had ad libitum access to food and water. Body weight measurements were taken weekly, while food and water intake were recorded daily throughout the study.

At the end of the experiments, the rats were anesthetized with sodium phenobarbital at a dose of 50 mg/kg body weight and euthanized by thoracotomy. Blood, liver and AT were collected. The whole AT sample and one portion of the liver were immediately frozen in liquid nitrogen and stored at −80 °C until analysis. The remaining liver portion was preserved in 10% formalin for histological assessment.

Blood samples were centrifuged at 3000× *g* for 15 min to obtain serum, which was then stored at −20 °C until further analysis.

### 4.4. Serum Measurements

Fasting glucose, TG, total cholesterol, and high-density lipoprotein cholesterol (HDL-c) were measured using commercial assay kits (Biosystem Laboratories, Barcelona, Spain) and an automated A15 spectrophotometer following the manufacturer’s instructions.

To determine serum FFAs, the methodology reported by Falholt et al. [45] was followed and the results were expressed in µg of palmitic acid equivalents. To generate a calibration curve, a diluted standard solution of palmitic acid was used (SIGMA Co., St. Louis, MO, USA).

Insulin levels were determined using a rat insulin enzyme-linked immunosorbent assay (ELISA) kit (Millipore, Burlington, MA, USA) according to the manufacturer’s instructions. A spectrophotometer (Spectronic^®^ 20, GenesysTM, Spectronic Instruments, Scottsdale, AZ, USA) was used.

For insulin resistance (IR) assessment, the HOMA-IR index was calculated with the following equation:

HOMA-IR = [FI × FG/405](3)

where FI corresponds to fasting insulin (µU/mL) and FG to fasting glucose (mg/dL).

Additionally, the QUICKI was calculated using the following equation:

QUICKI = 1/[log (fasting insulin, µU/mL) + log (fasting glucose, mg/dL)](4)

Higher QUICKI values indicate greater insulin sensitivity, while lower QUICKI values indicate insulin resistance.

### 4.5. Hepatic Lipid Content and Steatosis Evaluation

The hepatic TG content was determined according to the method described by Folch et al. [46]. Hepatic lipids were extracted from tissue homogenates using a chloroform:heptane:methanol (1:1:1, *v*/*v*/*v*) solvent system prepared in 50 mM phosphate buffer (pH 7.0). TG concentrations were quantified using commercial enzymatic assay kits (Biosystems Laboratories, Barcelona, Spain) following the manufacturer’s instructions. Hepatic TG content was expressed as mg of TG/g liver tissue.

For steatosis evaluation, samples preserved in formalin were embedded in paraffin, sectioned at 4–5 μm thickness, and stained with hematoxylin and eosin (H&E). Five images per section were captured, with ten fields analyzed per image. Histological evaluation was performed by J.L.G. The severity of hepatic steatosis was assessed using a semi-quantitative scoring system as follows: grade 0 = <5% steatosis (no significant fat accumulation), grade 1 (mild) = 5–33% of hepatocytes affected, grade 2 (moderate) = 34–66% of hepatocytes affected, and grade 3 (severe) = >66% of hepatocytes affected.

### 4.6. OS Markers Assessment

The OS markers were evaluated in the serum, liver, and AT. For the liver and AT, pulverized, frozen samples were homogenized in 50 mM phosphate buffer (pH 7.0) containing 0.5 mM EDTA. Protein concentrations in both serum and tissue homogenates were determined using the Bradford method [47].

Lipid oxidation was estimated through MDA concentration using the method reported by Rocha-Guzmán et al. [48]. The results were expressed as micromoles of MDA equivalents per mg of protein.

The methodology described by Sastry et al. [49] was used to measure the levels of end-products of NO_end-PD_. The results were expressed as µM/mg of protein.

The methodology reported by Sinha et al. [50] was employed to measure the activity of CAT, where the amount of enzyme that decomposes 1 μmol of H_2_O_2_ per min is defined as one unit (U) of CAT activity.

The methodology reported by Ukeda et al. [51] was used for SOD activity evaluation. The activity of SOD was quantified by the inhibition degree of blue tetrazolium chloride (NBT) reduction by the enzyme; one unit (U) is defined as the amount of enzyme necessary to suppress reduction in NBT by 50%.

### 4.7. Statistical Analysis

Data are presented as mean ± standard error (SE) or Median (Min–Max). The Shapiro–Wilk test was used to evaluate data distribution. For parametric data, a one-way analysis of variance (ANOVA) with Tukey’s post hoc test was performed. For non-parametric data, the Kruskal–Wallis test was used. The significance level was established with a *p* value < 0.05.

Statistical analyses were conducted using IBM SPSS Statistics for Windows, version 20 (IBM Corp., Armonk, NY, USA).

## 5. Conclusions

The present study demonstrates that chronic consumption of *Physalis philadelphica* husk infusion attenuated body weight gain and improved selected metabolic and oxidative stress parameters in diet-induced obese rats, including fasting glucose, circulating free fatty acids, and markers of oxidative stress. However, no significant effects were observed on insulin concentrations, HOMA-IR, QUICKI, hepatic steatosis, or several serum lipid parameters. These findings suggest that *P*. *philadelphica* husk infusion may contribute to the modulation of specific metabolic alterations associated with obesity, potentially through its antioxidant properties, rather than demonstrating a comprehensive anti-obesity effect. Therefore, the present results should be considered as preclinical evidence supporting the potential metabolic benefits of this traditionally consumed infusion. Further studies are required to elucidate its molecular mechanisms of action, evaluate additional pharmacological endpoints related to obesity, and to confirm its efficacy and safety in clinical settings.

## Figures and Tables

**Table 1:** Chemical characterization and determination of detection limits for the validation of the husk infusion of *Physalis philadelphica*.

No.	Compound	Transitions	RT (min)	LR (ng/mL)	Regression Equation	CE (*r*^2^)	LOD (ng/mL)	LOQ (ng/mL)	Husk Infusion (ng/mg of Lyophilized Infusion)
1	Gallic acid	169.15 > 125.05, 79.07	1.2	0–20	y = 10.275x − 10509	0.9973	0.03	0.09	1.30 ± 0.05
2	Protocatechuic acid	153.1 > 109.0, 91.04	2.1	0–24	y = 9.3208x − 6684.1	0.9976	0.07	0.21	74.0 ± 10.0
3	Chlorogenic acid	137.0 > 93.0	3.3	0–20	y = 8.8799x − 100229	0.9983	0.3	0.9	0.30 ± 0.01
4	Vanillic acid	167.1 > 123.0	3.7	0–20	y = 6.6925x	0.9640	0.01	0.03	4.10 ± 0.01
5	Caffeic acid	179.1 > 135.08, 89.09	3.9	0–32	y = 8.0015x − 4441.1	0.9986	0.02	0.06	1.01 ± 0.30
6	Epicatechin	289.1 > 245.1	4.3	0–20	y = 3 × 10^−6^x − 0.0719	0.9995	0.003	0.009	Traces
7	Ferulic acid	193.2 > 134.0, 178.07	5.7	0–32	y = 9.4918x + 410.52	0.9949	0.05	0.015	1.00 ± 0.10
8	Rutin	609.2 > 300.2	5.8	0–20	y = 2 × 10^−6^x − 0.3595	0.9704	0.003	0.01	0.02 ± 0.00
9	Kaempferol-3-O-Glc	447.3 > 255.1	6.7	0–20	y = 6 × 10^−6^x − 0.0088	0.9947	0.002	0.006	Traces
10	Quercetin	301.2 > 151.0	8.4	0–20	y = 4 × 10^−6^x − 0.1737	0.9898	0.001	0.003	2.10 ± 0.30
11	Luteolin	285.2 > 133.0	8.5	0–20	y = 2 × 10^−6^x − 0.23	0.9834	0.002	0.006	0.02 ± 0.00
12	Naringenin	271.2 > 151.4, 119.06	9.2	0–20	y = 2 × 10^−6^x − 0.144	0.9988	0.001	0.003	Traces

Determinations were carried out in duplicate. Values are means ± standard errors. RT: Retention time; CE: Correlation coefficient; LR: Linear range; LOD: Limit of detection; LOQ: Limit of quantification.

**Table 2:** Concentrations of end products of nitric oxide (NO_end-PD_) and malondialdehyde (MDA) in serum, liver, and adipose tissue in obese rats that received a husk infusion of *Physalis philadelphica*.

Sample	Serum (µM/mg Protein)		Liver (µM/mg Protein)		Adipose Tissue (µM/mg Protein)	
	NO_end-PD_	MDA	NO_end-PD_	MDA	NO_end-PD_	MDA
Healthy control	0.21 ± 0.01 *	1.26 ± 0.1 *	0.23 ± 0.00 *	1.06 ± 0.1 *	0.40 ± 0.05 *	0.79 ± 0.2 *
Obese control	0.52 ± 0.04 ^†^	7.2 ± 1.2	0.45 ± 0.02	2.83 ± 0.33 ^†^	0.74 ± 0.04	3.99 ± 0.23 ^†^
Husk infusion	0.35 ± 0.0 **	5.8 ± 1.0 **	0.32 ± 0.06 **	1.98 ± 0.27 **	0.72 ± 0.06 **	2.47 ± 0.24 **

Values are expressed as mean ± standard error. * Indicates a significant difference (*p* < 0.05) between the healthy control and the obese control group; ** indicates *p* < 0.05 between healthy rats and the husk infusion group; ^†^ indicates a significant difference (*p* < 0.05) between the obese control group and the husk infusion group. Significance was determined using the Kruskal–Wallis test.

## Data Availability

The original contributions presented in this study are included in the article and Supplementary Materials, including information regarding the sample size calculation and detailed information on the ethical procedures for animal experimentation. Further inquiries can be directed to the corresponding authors.

## Supplementary Materials

The following supporting information can be downloaded at: https://www.mdpi.com/article/10.3390/ijms27146500/s1. [19,52,53,54] are cited in the Supplementary Materials.

## Abbreviations

The following abbreviations are used in this manuscript.

TG	Triglycerides
AT	Adipose tissue
FFAs	Free fatty acids
IR	Insulin resistance
HOMA-IR	Homeostatic Model Assessment
QUICKI	Quantitative Insulin Sensitivity Check Index
OS	Oxidative stress
SOD	Superoxide dismutase
CAT	Catalase
MDA	Malondialdehyde
NO_end-PD_	Nitric oxide end products
