# Supplementary material for: Effects of Physalis philadelphica Lam. Husk Infusion on Obesity and Associated Metabolic Disturbances in an Experimental Model
Source: Int J Mol Sci. 2026 Jul 22;27(14):6500. doi: 10.3390/ijms27146500 (PMC13410684; doi:10.3390/ijms27146500)
Supplement: Supplementary file 1 [file ijms-27-06500-s001.zip › ijms-4329912-supplementary.pdf]

Supplementary Materials

Male Wistar rats (N=32), aged twelve weeks and weighing 220 ± 20 g (Universidad Autónoma de México, Campus Juriquilla, Querétaro, México) were maintained in an animal laboratory with 12-12 h light-dark cycle and 26 ± 1° C of temperature. A one-week acclimatization period was established before initiating the experiment. Throughout this acclimatization phase and during the experiment, the rats had unrestricted access to rodent lab chow 5001 (Purina®, Québec, Canada).

The experimental unit was a single animal.

Criteria for including or excluding animals during the experiment and for excluding data points during the analysis followed the guidelines established in the Mexican Official Norm (NOM-062-ZOO-1999). Animals showing signs of pain or illness were to be excluded, and if recovery was not possible, euthanasia would be performed. In our study, no such cases were observed; therefore, all animals completed the experiment."

For all analyses, the number of rats per group was as follows:

Healthy control (n = 8)

Obese control (n = 8)

Obese + husk infusion (n = 8)

The sample size calculation was based on the following equation for serial incidences [52]:

$$X = N / ((A/100) \times (B/100) \times (C/100)...).$$

Where:

X=Final number of animals required

N=Minimum number needed to meet the objectives proposed in the project

A=100 minus the percentage of incidence 1

B=100 minus the percentage of incidence 2

C=100 minus the percentage of incidence 3, and so on.

Considering a minimum of four individuals for similar animal models [53], an incidence rate of 25% as reported in the literature [19], and ethical considerations aligned with the principles of the 3Rs (Replacement, Reduction, and Refinement) [54], a sample size of eight rats per group was selected to ensure the minimum number necessary to obtain meaningful data.

Randomization was performed based on initial body weight to ensure homogeneous groups under baseline conditions prior to obesity induction. To minimize potential confounders—such as the order

of treatments and measurements, or animal and cage location—each animal was individually identified using ear tags and appropriately labeled cages were used to maintain consistent tracking throughout the study.

Dr. Claudia I. Gamboa Gómez, the corresponding author, was aware of group allocation at all stages of the experiment, including allocation, conduct of the experiment, outcome assessment, and data analysis.

All outcome measures assessed were biochemical and molecular markers. The primary outcome measure was the change in these parameters compared to the control groups.
